# Relationships between Interaction Energy and Electron Density Properties for Homo Halogen Bonds of the [(A)*_n_*Y–X···X–Z(B)*_m_*] Type (X = Cl, Br, I)

**DOI:** 10.3390/molecules24152733

**Published:** 2019-07-27

**Authors:** Maxim L. Kuznetsov

**Affiliations:** Centro de Química Estrutural, Instituto Superior Técnico, Universidade de Lisboa, Avenida Rovisco Pais, 1049-001 Lisbon, Portugal; max@mail.ist.utl.pt; Tel.: +351-218-419-236

**Keywords:** bond critical point properties, interaction energy, bond energy, bond strength, density functional theory, electron density, energy density, halogen bond, QTAIM

## Abstract

Relationships between interaction energy (E_int_) and electron density properties at the X···X bond critical point or the d(X···X) distance were established for the large set of structures [(A)*_n_*Y–X···X–Z(B)*_m_*] bearing the halogen bonds Cl···Cl, Br···Br, and I···I (640 structures in total). The best estimator of E_int_ is the kinetic energy density (G_b_), which reasonably approximates the whole set of the structures as −E_int_ = 0.128G_b_^2^ − 0.82G_b_ + 1.66 (R^2^ = 0.91, mean absolute deviation 0.39 kcal/mol) and demonstrates low dispersion. The potential and kinetic energy densities, electron density, and the d(X···X) distance behave similarly as estimators of E_int_ for the individual series Cl···Cl, Br···Br, and I···I. A number of the E_int_(property) correlations are recommended for the practical application in the express estimates of the strength of the homo-halogen bonds.

## 1. Introduction

Halogen bonds (XBs) belong to the most important type of non-covalent interactions (apart from hydrogen bonds) [1,2,3,4,5,6,7,8,9,10,11]. XB is usually formulated as an interaction of the Y–Hal···X–Z type where Hal is a halogen atom (Lewis acid, XB donor) bearing a region with the positive electrostatic potential directed toward the X atom (the co-called σ-hole) and X is a donor of electron density (Lewis base, XB acceptor) (X = N, O, S, Se, Hal, Hal^−^, etc.). Halogen bonds play a very important role in molecular recognition and crystal engineering, synthesis of functional solid materials [12,13,14,15,16,17,18,19,20,21,22], and drug design [23,24,25]. They have a broad application in the tuning of useful functional properties such as redox, magnetic [26,27], or catalytic [28,29,30] properties, and nonlinear optical (NLO) activity [31,32]. Non-covalent interactions, including XB, are involved in the control of various biochemical processes participating in the organization of the secondary, tertiary, and quaternary protein structures [33,34,35,36,37].

One of the most important characteristics of XB controlling its functional properties is the halogen bond energy (E_XB_). Experimental determination of E_XB_ is usually associated with significant technical issues [38,39], while its direct theoretical calculations are not always possible or reliable (e.g., for the intramolecular XBs or molecular associates involving several non-covalent interactions, Watson-Crick base pairs being among them). One of the most attractive and sometimes the only possible methods of the approximate express estimate of the bond energy for weak interactions is the application of appropriate E_XB_(property) correlations [40,41,42,43,44,45,46,47,48,49,50,51,52,53,54,55,56,57,58,59,60,61,62,63,64,65,66,67,68,69,70,71,72,73,74,75,76,77,78,79,80,81,82,83,84,85,86,87]. This method has got an impulse after the publication of an article by Espinosa, Molins, and Lecomte [88]. It was found that the interaction energy (E_int_) of hydrogen bonds of the X–H···O type correlates with the potential energy density at the bond critical point (BCP) of the electron density distribution, V_b_, as Equation (1)

E_int_ ≈ 0.5V_b_ (1)

(the co-called EML formula, the interaction energy between two fragments A and B is defined as E_int_ = E_A–B_ − E_{A}_ − E_{B}_, where E_A–B_ is energy of the fully optimized molecule A–B, E_{A}_, and E_{B}_ are energies of the fragments A and B with unrelaxed geometries corresponding to the equilibrium structure A–B).

Later, similar relationships between E_int_ and V_b_ or kinetic energy density at BCP (G_b_) were deduced for the FH···FR hydrogen bonds [89,90] (Equation (2)) and for the Y–Hal···Z(B)*_m_* halogen bonds (Hal = Cl, Br, I; Z = N, S, O, C) [79] (Equations (3)–(5)).

E_int_ ≈ −0.429G_b_ (2)

E_int_ ≈ 0.49V_b_ ≈ −0.47G_b_, for Hal = Cl (3)

E_int_ ≈ 0.58V_b_ ≈ −0.57G_b_, for Hal = Br (4)

E_int_ ≈ 0.68V_b_ ≈ −0.67G_b_, for Hal = I. (5)

Since the publication of the EML formula, numerous but usually not justified attempts to apply this relationship to different types of non-covalent interactions were undertaken. However, it was shown that these equations may not be universal, and their validity may be restricted only to the type of interactions for which they were deduced [91,92]. Thus, there is a great practical need to establish the reliable E_int_(property) relationships for the most important types of non-covalent interactions others than the X–H···O, FH···FR, and Y–Hal···Z(B)*_m_* ones.

Recently, the author started the project aimed to establish the E_int_(property) relationships for various types of non-covalent interactions. In the first publication of this research cycle [92], the halogen bonds of the [(A)*_n_*Z–Y···X]^−^ type (X, Y = F, Cl, Br; Z = F, Cl, Br, I, C, N, O, H, S, P, Si, B, totally 441 structure) formed upon interaction of the neutral fragment (A)*_n_*Z–Y and the halide anion X^−^ were considered. It was shown that the E_int_(property) correlations are different for each particular type of the Y···X^−^ interaction and they are also different from Equations (1)–(4). Several E_int_(property) relationships practically important for express estimates of the interaction energy were recommended for each series of these structures.

In this work, the E_int_(property) relationships are analyzed for the homo-halogen bonds of the [(A)*_n_*Y–X···X–Z(B)*_m_*] type formed upon the interaction of two neutral fragments (A)*_n_*Y–X and X–Z(B)*_m_* (X = Cl, Br, I; Y, Z = F, Cl, Br, C, N, O, H, S, P, Si, B). For the first time, the E_int_(property) relationships were deduced for the Cl···Cl, Br···Br, and I···I homo-XBs based on a large, statistically significant set of structures (640 structures in total).

## 2. Computational Details

Full geometry optimization and energy calculations of all structures were carried out at the DFT level of theory by using the M06-2X functional [93] with the help of the Gaussian 09 [94] program package applying the tight optimization criteria and ultrafine integration grid. Cartesian d and f basis functions (6d, 10f) were used in all calculations. The M06-2X function reasonably describes weak dispersion forces and is widely and successfully used for the treatment of structures with non-covalent interactions. It was shown by Kozuch and Martin [95] for a set of 51 structures bearing XBs that the root-mean-square deviation, the mean signed error, and the maximum error of dissociation energy for M06-2X are 0.43, 0.01, and 1.58 kcal/mol, respectively, relative to the CCSD(T) method. Additionally, it was shown [92] that the E_int_(V_b_) correlation for structures of the [(A)*_n_*Z–Y···F]^−^ type (Y = F, Cl, Br) at the M06-2X level of theory has similar parameters as that for the MP4, CCSD, and CCSD(T) methods (see Figure 7A in [90]).

Molecular systems of the medium and large dimension are usually computationally treated using double zeta quality basis sets with corresponding diffuse and polarization functions. Therefore, to ensure that the results of this work may be successfully transferred to larger molecular systems, the 6-31+G* basis set was used for structures of the Cl···Cl and Br···Br types and the all electron DZP basis set [96,97,98] was applied for all atoms of the I···I structures. Previously [92], it was shown that the 6-31+G* basis set performs similarly to the much more extended triple zeta basis set 6-311++G(3df,3pd) in the direct calculations of E_int_ and the electron density based properties of the [(A)*_n_*Z–Y···F]^−^ structures. Coefficients of the E_int_(V_b_) correlations obtained for these two basis sets are also similar (see Figures 5C, 6C, and 7D in [90]). Meanwhile, the effect of the computational method and basis set is worthwhile to investigate but this should be the subject of separate work.

The Hessian matrix was calculated for all optimized structures to prove the location of correct minima. No symmetry operations were applied during the calculations. The stability test was performed and the stable solutions were achieved for all structures using the keyword STABLE(OPT). BSSE was corrected using the counterpoise (CP) method [99,100]. A detailed discussion of the BSSE effect for each Cl···Cl, Br···Br, and I···I series is provided in the Supplementary Material. The topological analysis of the electron density distribution was performed with the help of the AIM method developed by Bader [101] using the AIMAll program [102].

## 3. Computational Models

For this study, structures [(A)*_n_*Y–X···X–Z(B)*_m_*] (X = Cl, Br, I; Y, Z = F, Cl, Br, C, N, O, H, S, P, Si, B) bearing the homo-halogen bonds Cl···Cl, Br···Br, and I···I and formed upon interaction of two neutral fragments (A)*_n_*Y–X and X–Z(B)*_m_* were selected as computational models (640 structures in total, among them 210 structures of the Cl···Cl type, 216 structures of the Br···Br type, and 214 structures of the I···I type). In this work, the fragment (A)*_n_*Y–X (at the left side of the complex formula) corresponds to the XB donor and the fragment X–Z(B)*_m_* (at the right side of the complex formula) corresponds to the XB acceptor.

For each type of halogen bonds, three series were considered, i.e., [(A)*_n_*Y–X···X–F], [(A)*_n_*Y–X···X–H], and [F–X···X–Z(B)*_m_*]. In the first two series, the XB donor part is variable while the XB acceptor part is fixed with the Z(B)*_m_* group being either a strong electron acceptor (Z(B)*_m_* = F) or an electron donor (Z(B)*_m_* = H). Series of the structures with even stronger electron donor groups Z(B)*_m_* = CMe_3_, CHMe_2_, or CH_3_ were also calculated. However, in most of cases, secondary X···H–C interactions were found and, therefore, the individual X···X halogen bonds cannot be isolated for the analysis.

In the third series, [F–X···X–Z(B)*_m_*], the XB acceptor part is variable whereas the XB donor part is fixed ((A)*_n_*Y = F). Most of the attempts to calculate similar series with an electron donor group (A)*_n_*Y = H failed because the σ-hole at the terminal X atom of the XB donor is not sufficiently pronounced in this case, and other interactions prevail in the resulting structures.

Ninety various groups (A)*_n_*Y or Z(B)*_m_* were considered to analyze the effect of the second-order atoms Y and Z and remote groups A and B as well as the effects of the orbital hybridization and the oxidation state of the Y and Z atoms on the E_int_(property) correlations. The groups A and B vary from strong electron donors to strong electron acceptors to ensure a broad interval of the interaction energies within each series (see Table S1 in the Supplementary Material for the complete list of the calculated structures).

**Figure 1:**
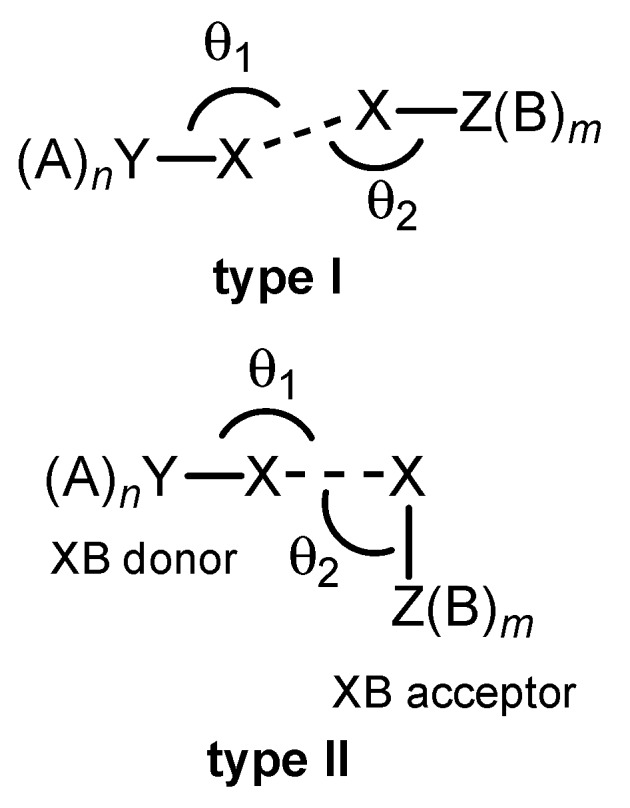
Two types of the halogen bond geometries.

There are two typical geometries of the fragments with halogen bonds [103]. The first one is characterized by the similar (or equal) angles θ (θ_1_ ≈ θ_2_) (type I, Figure 1). The second geometry corresponds to θ_1_ ≈ 180° and θ_2_ ≈ 90° (type II, Figure 1). In this work, only structures of type II are discussed since they are typically more stable than structures of type I. Indeed, 54 structures of type II and only 14 structures of type I were successfully optimized for the [(A)*_n_*Y–Cl···Cl–H] series (90 various (A)*_n_*Y groups were considered, Table S1).

Only structures which have no other contacts shorter than the sum of van der Waals radii between the (A)*_n_*Y–X and X–Z(B)*_m_* fragments, apart from the X···X one, are included in the analysis. Additionally, only structures with θ_2_ ≥ 70° were analyzed because, in those with θ_2_ < 70°, the X···Z interaction may play a significant or even a predominant role compared to the X···X interaction (Sixty eight structures of the [F–X···X–Z(B)m] type with the X···Z contact shorter the sum of van der Waals radii but with θ2 ≥ 70° and with no other weak interactions were included in the analysis since no BCPs for the X···Z contacts were found). Effect of the angle θ_2_ on the correlations under study is discussed in Section 4.6. “Effect of angle θ_2_”.

Further in this work, the series [(A)*_n_*Y–Cl···Cl–Z(B)*_m_*], [(A)*_n_*Y–Br···Br–Z(B)*_m_*], and [(A)*_n_*Y–I···I–Z(B)*_m_*] are called “large”, while the series [(A)*_n_*Y–Cl···Cl–H], [(A)*_n_*Y–Cl···Cl–F], [F–Cl···Cl–Z(B)*_m_*], [(A)*_n_*Y–Br···Br–H], [(A)*_n_*Y–Br···Br–F], [F–Br···Br–Z(B)*_m_*], [(A)*_n_*Y–I···I–H], [(A)*_n_*Y–I···I–F], and [F–I···I–Z(B)*_m_*] are called “small” series.

## 4. Results and Discussion

The calculated X···X interaction energy with the BSSE correction in the whole set of the structures varies from 0.34 to −9.24 kcal/mol. All three types of halogen bonds, Cl···Cl, Br···Br, and I···I, have comparable strengths. The dispersion of E_int_ increases along the row X = Cl < Br < I (−0.18 to −5.01, 0.08 to −7.10, and 0.34 to −9.24 kcal/mol, respectively). The Cl···Cl interaction is slightly weaker than the corresponding Br···Br and I···I bonds for the [(A)*_n_*Y–X···X–H] and [F–X···X–Z(B)*_m_*] series (Table S2 in the Supplementary Material). The halogen bonds in the series [F–X···X–Z(B)*_m_*] are the strongest ones while the XBs in the series [(A)*_n_*Y–X···X–F] are typically the weakest bonds.

In the next sections, the E_int_(property) relationships are considered for each estimator [i.e., the electron density, ρ_b_, its Laplacian, ∇^2^ρ_b_, the curvature of ρ(**r**) which is parallel to the bond path direction (positive), λ_||,b_, potential, kinetic, and total energy densities, V, G_b_, and H_b_ and the X···X distance, d(X···X)] within the whole set and various series of the structures.

### 4.1. Whole Set of Structures

**Figure 2:**
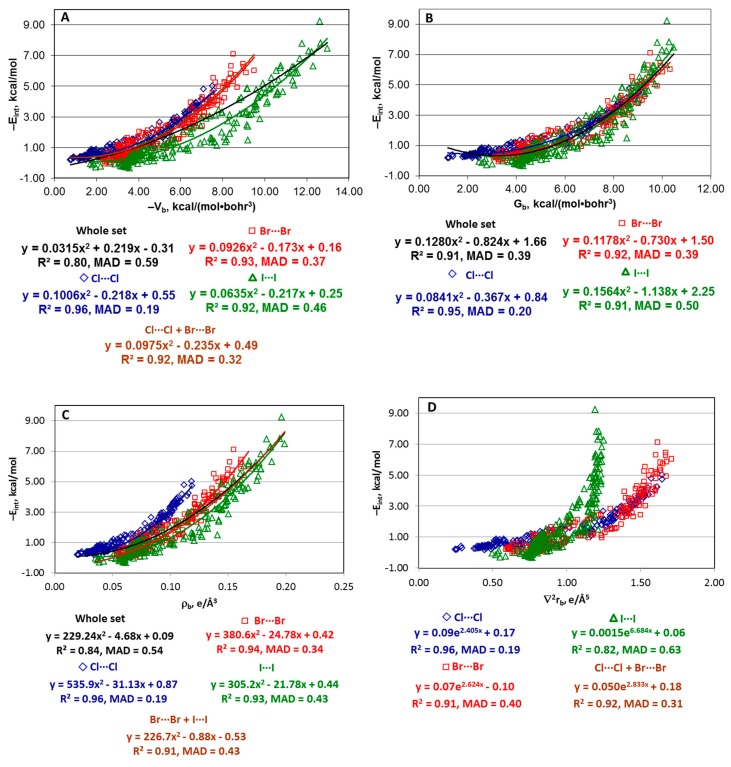

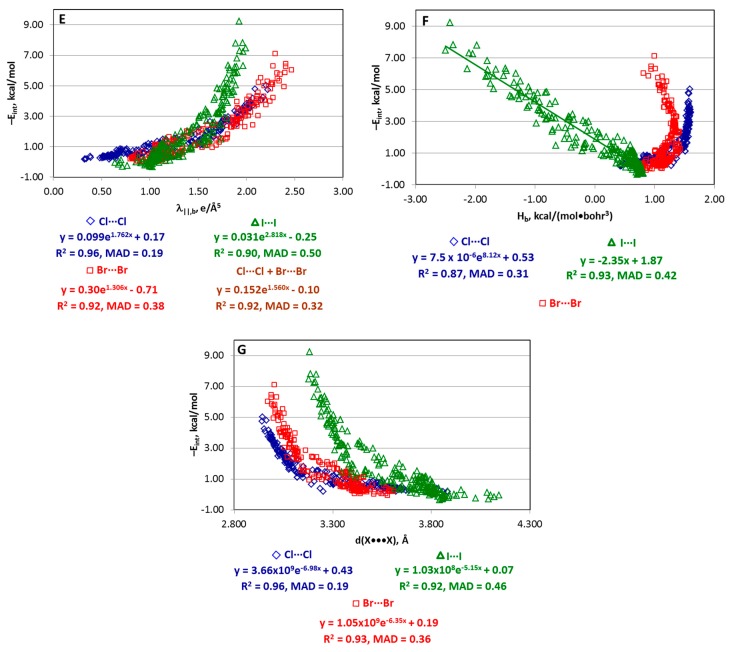
Plots of −E_int_ against −V_b_ (**A**), G_b_ (**B**), ρ_b_ (**C**), ∇^2^ρ_b_ (**D**), λ_||,b_ (**E**), H_b_ (**F**), and d(X···X) (**G**) for the whole set of the structures [(A)*_n_*Y–X···X–Z(B)*_m_*] and the “large” series [(A)*_n_*Y–Cl···Cl–Z(B)*_m_*], [(A)*_n_*Y–Br···Br–Z(B)*_m_*], and [(A)*_n_*Y–I···I–Z(B)*_m_*].

**Potential energy density at BCP (V_b_).** A rough correlation between E_int_ and the potential energy density at the X···X BCP is observed for the whole set of the structures (Figure 2A). Very curiously, this dependence is obviously nonlinear and may be approximated with the similar quality by either a quadratic or an exponential function −E_int_ = 0.0315V_b_^2^ − 0.219V_b_ − 0.31 or −E_int_ = 3.67e^−0.09Vb^ − 4.19. Such behavior is unusual since most of the E_int_(V_b_) correlations reported in literature obey a linear law. The R^2^ values (0.80) indicate that the correlations are of rather poor quality. Meanwhile, the mean absolute deviation (MAD) for these fittings is ~0.60 kcal/mol. This value is well within the typical accuracy of the DFT methods (several kcal/mol) and this is almost three times less than the average interaction energy for this structural set (1.72 kcal/mol).

Thus, in the case of structures [(A)*_n_*Y–X···X–Z(B)*_m_*] with the homo-halogen bonds formed upon interaction of the neutral fragment, E_int_ may be roughly estimated from V_b_ using a single formula for all X = Cl, Br, and I. This situation is very different from that found recently for the set of anionic structures [(A)*_n_*Z–Y···X]^−^ (Y, X = F, Cl, Br) [92]. In the latter case, the E_int_(V_b_) relationship is much more sensitive to the nature of the interacting X and Y atoms, and there is no single dependence which could approximate E_int_ through V_b_ for the whole set.

**Kinetic energy density at BCP (G_b_).** The quadratic and exponential relationships between E_int_ and G_b_ (−E_int_ = 0.1280G_b_^2^ − 0.824G_b_ + 1.66 and − E_int_ = 0.144e^0.39Gb^ − 0.13) display significantly lower dispersion and have noticeably better quality than the E_int_(V_b_) dependence (Figure 2B). The R^2^ value is quite reasonable (0.91) and MAD is 0.39 and 0.40 kcal/mol that is more than four times lower than the average interaction energy for this structural set (1.72 kcal/mol). Thus, the kinetic energy density at BCP is a better estimator of E_int_ compared to V_b_, and a single function may be used for the reasonable approximation of interaction energy for the whole set of structures bearing the homo-halogen bonds Cl···Cl, Br···Br, and I···I. Meanwhile, both E_int_(V_b_) and E_int_(G_b_) correlations found here, being strongly nonlinear, are qualitatively different from the EML formula and other relationships published in the literature for other types of non-covalent interactions (Equations (1)–(5)) [79,88,89,90].

**Electron density at BCP (ρ_b_).** The correlation of E_int_ against ρ_b_ is also clearly nonlinear that is different from most of the cases reported in the literature. Quality of the E_int_(ρ_b_) relationship is intermediate between the E_int_(V_b_) and E_int_(G_b_) dependencies (Figure 2C).

**Other estimators.** There are no single E_int_(property) trends for other estimators [∇^2^ρ_b_, λ_||,b_, H_b_, and d(X···X)] which could describe the whole set of structures. At least two various dependencies are clearly visible on each E_int_(property) plot (Figure 2D–G).

### 4.2. Series [(A)_n_Y–Cl···Cl–Z(B)_m_], [(A)_n_Y–Br···Br–Z(B)_m_], and [(A)_n_Y–I···I–Z(B)_m_]

**Potential energy density at BCP.** The E_int_(V_b_) correlations for each specific type of the halogen bond are of significantly better quality than those for the whole set in terms of both R^2^ (0.92–0.96) and MAD (0.18–0.45 kcal/mol) (Figure 2A). The Cl···Cl structures are clearly better described than the Br···Br or I···I structures. Both series [(A)*_n_*Y–Cl···Cl–Z(B)*_m_*] and [(A)*_n_*Y–Br···Br–Z(B)*_m_*] may be well approximated by a single quadratic or exponential function, the former performing slightly better with R^2^ = 0.92 and MAD = 0.32 kcal/mol. The [(A)*_n_*Y–I···I–Z(B)*_m_*] series lies below the series with the Cl···Cl and Br···Br bonds on the −E_int_(–V_b_) plot.

**Kinetic energy density at BCP.** The kinetic energy density behaves similarly to the potential energy density for the estimate of E_int_ within these series with the MAD values being only slightly worse (R^2^ = 0.92–0.96, MAD = 0.18–0.47 kcal/mol) (Figure 2B). However, G_b_ demonstrates lower dispersion compared to V_b_. Indeed, the E_int_(G_b_) relationships for all series [(A)*_n_*Y–Cl···Cl–Z(B)*_m_*], [(A)*_n_*Y–Br···Br–Z(B)*_m_*] and [(A)*_n_*Y–I···I–Z(B)*_m_*] have similar parameters despite the application of different basis sets (6-31+G* for the Cl···Cl and Br···Br structures and DZP for the I···I structures, although both these basis sets are of a double zeta quality). Meanwhile, the E_int_(V_b_) relationship for the iodine halogen bonds is quite different from those for the Cl···Cl and Br···Br structures. Thus, the application of G_b_ as an estimator of E_int_ is preferable over V_b_ also from this point of view.

**Electron density at BCP.** The E_int_(ρ_b_) relationships for these series are similar to E_int_(V_b_) and E_int_(G_b_) (Figure 2C). In contrast to V_b_, both series [(A)*_n_*Y–Br···Br–Z(B)*_m_*] and [(A)*_n_*Y–I···I–Z(B)*_m_*] may be approximated by a single function (R^2^ = 0.91, MAD = 0.43 kcal/mol), while the fitting for the [(A)*_n_*Y–Cl···Cl–Z(B)*_m_*] series has quite different parameters.

**Laplacian and curvature of electron density distribution at BCP.** Both E_int_(∇^2^ρ_b_) and E_int_(λ_||,b_) relationships for these series have similar features and are described by either quadratic or exponential function (Figure 2D,E). Quality of the approximations for the Cl···Cl series is similar to the V_b_, G_b_, and ρ_b_ estimators. However, both ∇^2^ρ_b_ and λ_||,b_ perform worse for the Br···Br and, in particular, I···I series. Both Cl···Cl and Br···Br series may be quite well treated by a single function, whereas the fitting parameters for the I···I series are very different. Various basis sets used for the Cl···Cl + Br···Br and I···I structures are conceivably responsible for this effect.

**Total energy density at BCP.** The E_int_(H_b_) relationships demonstrate different behavior for the Cl···Cl and Br···Br series, on one side, and for the I···I series, on the other side (Figure 2F). For the Cl···Cl series, the negative interaction energy increases with the enhancement of H_b_. This series may be approximated by a single exponential function but with rather poor quality for this type of the halogen bond (R^2^ = 0.87 and MAD = 0.31 kcal/mol). For the Br···Br series, the E_int_(H_b_) function is not well-defined. Finally, for the I···I structures, −E_int_ increases with the decrease of H_b_. The −E_int_(H_b_) relationship is nearly linear with R^2^ = 0.93 and MAD = 0.42 kcal/mol and may be used for the estimate of E_int_ for this type of halogen bonds.

**Internuclear distance d(X···X).** The −E_int_(d(X···X)) dependencies are exponential with rather different parameters for the Cl···Cl, Br···Br, and, in particular, I···I structures (Figure 2G). The quality of this estimator is similar to V_b_, G_b_, and ρ_b_.

### 4.3. Series [(A)_n_Y–X···X–H], [(A)_n_Y–X···X–F], and [F–X···X–Z(B)_m_]

**Figure 3:**
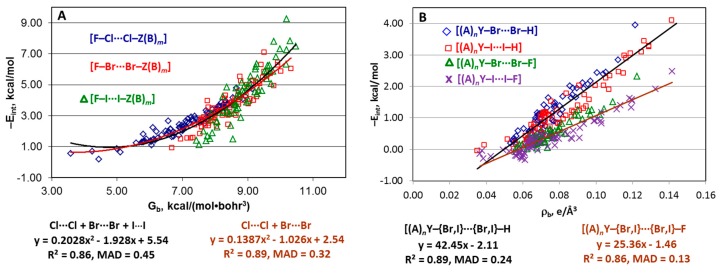
Plots of −E_int_ against G_b_ for the series [F–X···X–Z(B)*_m_*] (**A**) and against ρ_b_ for the series [(A)*_n_*Y–{Br,I}···{Br,I}–H] and [(A)*_n_*Y–{Br,I}···{Br,I}–F] (**B**).

Typically, there are no statistically meaningful trends describing these series (Figure S1 in the Supplementary Material). Several dependencies corresponding to the “small” series with quite different parameters are clearly visible on the E_int_(property) plots. The best E_int_(G_b_) approximation was found for the [F–X···X–Z(B)*_m_*] series with R^2^ = 0.86 and MAD = 0.44 kcal/mol (Figure 3A). The E_int_(ρ_b_) function also reasonably describes each of the pairs “[(A)*_n_*Y–Br···Br–H] + [(A)*_n_*Y–I···I–H] and “[(A)*_n_*Y–Br···Br–F] + [(A)*_n_*Y–I···I–F] (Figure 3B) but the fitting parameters for the corresponding Cl···Cl series are quite different. This situation demonstrates that the E_int_(property) relationships significantly depend on the nature of the interacting atoms X.

### 4.4. “Small” Series

**Figure 4:**
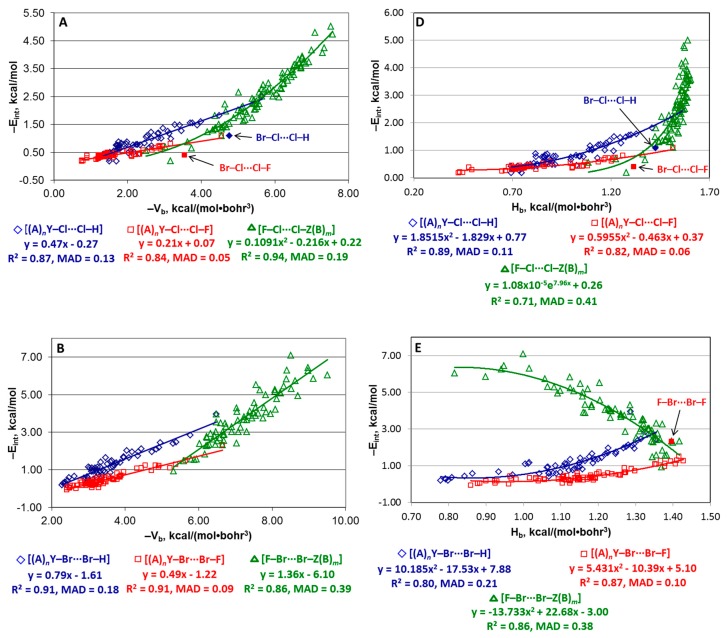

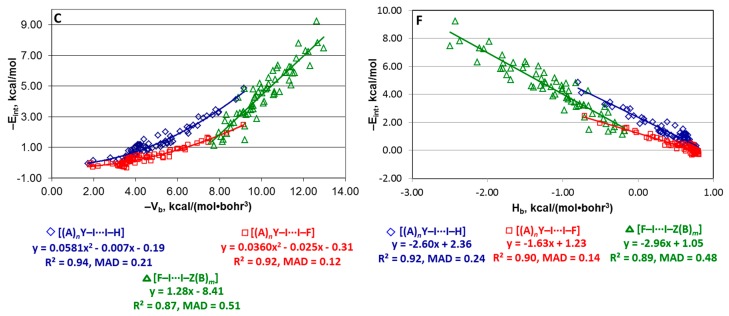
Plots of −E_int_ against –V_b_ (**A**–**C**) and H_b_ (**D**–**F**) for the “small” series. Points corresponding to structures Br–Cl···Cl–H, Br–Cl···Cl–F, and F–Br···Br–F are not included in the fitting in parts **A**, **D**, and **E**.

**Potential energy density at BCP.** The E_int_(V_b_) relationship for each of nine “small” series may be reasonably approximated by a linear function. The quadratic fitting is noticeably better than the linear one only for the [F–Cl···Cl–Z(B)*_m_*], [(A)*_n_*Y–I···I–H], and [(A)*_n_*Y–I···I–F] series (Figure 4A–C). The points corresponding to Br–Cl···Cl–H and Br–Cl···Cl–F are clearly out of the trends and they were excluded from the further analysis. Analysis of the correlations indicates the following.

First, the slope and negative interception of the −E_int_(−V_b_) relationships for the [F–Cl···Cl–Z(B)*_m_*], [F–Br···Br–Z(B)*_m_*], and [F–I···I–Z(B)*_m_*] series are significantly higher than for the other series, whereas those for the [(A)*_n_*Y–Cl···Cl–F], [(A)*_n_*Y–Br···Br–F], and [(A)*_n_*Y–I···I–F] structures are the lowest.

Second, quality of the dependencies for the “small” series is usually worse than for the “large” ones in terms of R^2^. However, the MAD values for the [(A)*_n_*Y–{Cl,Br,I}···{Cl,Br,I}–{H,F}] “small” series (0.05–0.21 kcal/mol) are significantly lower than for the corresponding “large” series (0.18–0.45 kcal/mol). The estimate of E_int_ for the structures [F–Br···Br–Z(B)*_m_*] and [F–I···I–Z(B)*_m_*] is preferable using equations for the “large” series because both MAD and R^2^ parameters are worse for these “small” series than for the corresponding “large” ones.

Third, among all “small” series, only one, [(A)*_n_*Y–Cl···Cl–H], is described by an equation similar to the EML formula (E_int_ = 0.47V_b_ + 0.27). Equations (3)–(5) obtained for the halogen bonds of the X···D type (X = Cl, Br, I; D = N, S, O, C) [79] are also not applicable to structures of the [(A)*_n_*Y–X···X–Z(B)*_m_*] type discussed in this work (except the same [(A)*_n_*Y–Cl···Cl–H] series).

**Total energy density at BCP.** For the Cl···Cl structures, the −E_int_(H_b_) function shows similar quadratic or exponential behavior for all three “small” series [(A)*_n_*Y–Cl···Cl–H], [(A)*_n_*Y–Cl···Cl–F] and [F–Cl···Cl–Z(B)*_m_*] (Figure 4D) although with quite different parameters: interaction energy −E_int_ increases with the enhancement of H_b_. For the Br···Br structures, the −E_int_(H_b_) relationship is of the same type for [(A)*_n_*Y–Br···Br–H] and [(A)*_n_*Y–Br···Br–F] (Figure 4E). However, −E_int_ increases with the reduction of H_b_ as a quadratic/exponential function for [F–Br···Br–Z(B)*_m_*] and as a linear function for the I...I structures (Figure 4F). The quality of all E_int_(H_b_) dependencies for the “small” series is worse than for the other estimators.

**Other estimators.** The main features of the E_int_(G_b_), E_int_(ρ_b_), E_int_(∇^2^ρ_b_), E_int_(λ_||,b_), and E_int_(d(X···X)) relationships for the “small” series are similar to the corresponding E_int_(V_b_) dependencies (Figure S2 in Supplementary Material). Laplacian describes the series [F–I···I–Z(B)*_m_*] particularly poor.

### 4.5. Estimate of E_int_ from an Integral of Electronic Virial over Interatomic Zero-Flux Surface (IAS)

**Figure 5:**
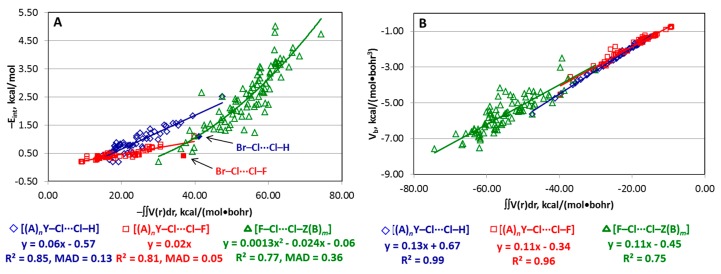
Plots of −E_int_ (**A**) and V_b_ (**B**) against (−/+)$\iint_{IAS}^{}V\left(r\right)dr$. Points corresponding to structures Br–Cl···Cl–H and Br–Cl···Cl–F are not included in the fittings in part **A**.

Recently, Romanova, Lyssenko, and Ananyev [104] reported that the integral of electronic virial V(**r**) over IAS ($\iint_{IAS}^{}V\left(r\right)dr$, **r** ∈ IAS) may be a better estimator of E_int_ than the potential energy density at BCP (V_b_). This result was obtained for a set of 50 structures with very different types of non-covalent interactions. Here, the quality of this estimator was verified for structures of the [(A)*_n_*Y–Cl···Cl–Z(B)*_m_*] type. The calculations indicated that the IAS integral of electronic virial field behaves similarly as V_b_ for the [(A)*_n_*Y–Cl···Cl–H] and [(A)*_n_*Y–Cl···Cl–F] series (compare Figure 4A and Figure 5A). However, the former estimator works much worse for the [F–Cl···Cl–Z(B)*_m_*] series providing unacceptably poor R^2^ value (0.77) and relatively high MAD (0.36 kcal/mol). Correspondingly, the E_int_($\iint_{IAS}^{}V\left(r\right)dr$) correlation for the whole series [(A)*_n_*Y–Cl···Cl–Z(B)*_m_*] is also significantly worse than the E_int_(V_b_) relationship with R^2^ = 0.89 *vs.* 0.96 and MAD = 0.28 vs. 0.18 kcal/mol.

The plot of V_b_ against $\iint_{IAS}^{}V\left(r\right)dr$ demonstrates a nice linear correlation for [(A)*_n_*Y–Cl···Cl–H] (R^2^ = 0.99), a quite good relationship for [(A)*_n_*Y–Cl···Cl–F] (R^2^ = 0.96) but a poor dependence for [F–Cl···Cl–Z(B)*_m_*] (R^2^ = 0.75) (Figure 5B). Thus, the integral of electronic virial over IAS cannot be recommended as an acceptable estimator of E_int_ at least for the structures bearing the Cl···Cl halogen bond.

### 4.6. Effect of Angle θ_2_

Although no BCP was found for the X_1_···Z contact in structures [(A)*_n_*Y–X_1_···X_2_–Z(B)*_m_*] included in the analysis, some of them have the angleX_1_X_2_Z (θ_2_) lower than 90°. This may point out some interaction between the atoms X_1_ and Z. To verify if this possible interaction affects the E_int_(property) dependencies, all structures were divided into three groups, i.e., those with θ_2_ ≥ 90°, 80° ≤ θ_2_ < 90°, and 70° ≤ θ_2_ < 80°. In the second and third groups, structures with the X_1_···Z distance shorter than the sum of van der Walls radii were also separated from those with the X_1_···Z distance longer than this sum.

**Figure 6:**
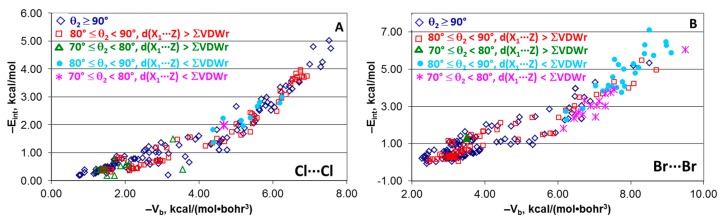

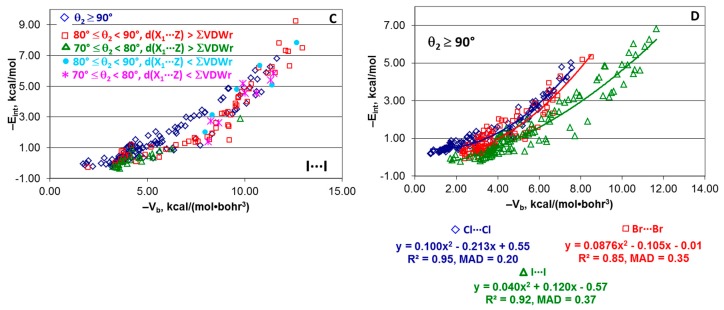
Plots of −E_int_ against −V_b_ for the “large” series of structures [(A)*_n_*Y–X···X–Z(B)*_m_*] with various angles θ_2_ (**A**–**C**) and distances X_1_···Z (**D**).

The E_int_(V_b_) dependencies for all these groups of structures are shown in Figure 6A–C. All structures fit very well the same trends within each series independently on the angle θ_2_ and the X_1_···Z distance. If considering only structures with θ_2_ ≥ 90°, R^2^ and MAD values are similar to those for the complete structural series except the Br···Br structures (compare Figure 2A and Figure 6D). In the latter case, exclusion of the structures with θ_2_ < 90° results in a much poorer R^2^ value of 0.85 compared to the complete set for which R^2^ = 0.93. All this indicates no effect of the angle θ_2_ and the X_1_···Z distance (within the selected ranges) on the E_int_(V_b_) dependencies. Similar results were obtained for all other estimators.

## 5. Final Remarks

In this work, the E_int_(property) correlations were established and analyzed for the first time for the large statistically significant sets of the homo-halogen bonds Cl···Cl, Br···Br, and I···I formed upon interaction of two neutral fragments at the M06-2X/6-31+G* (the Cl···Cl and Br···Br bonds) and M06-2X/DZP (the I···I bond) levels of theory. Electron density, its Laplacian, curvature of the electron density distribution, potential, kinetic, and total energy densities at BCP, integral of electronic virial over IAS and the d(X···X) internuclear distance were examined as estimators of the interaction energy. The correlations obtained in this work have a significant practical potential since they lead to interaction energies of the entire classes of the halogen bonds with the electron density distribution or even only the d(X···X) internuclear distance being in the hands. This is particularly important for the systems with multiple intermolecular non-covalent interactions or those bearing intramolecular XBs because a direct determination or calculation of E_int_ for such systems is an extremely difficult or often even impossible task. The following conclusions can be made.

First, the whole set of structures can be reasonably approximated by a single quadratic function −E_int_ = 0.128G_b_^2^ − 0.82G_b_ + 1.66 with R^2^ = 0.91 and MAD = 0.39 kcal/mol. For other estimators, no reasonable single correlation was found for the whole structural set.

Second, each of the series Cl···Cl, Br···Br, and I···I can be well described individually by the E_int_(V_b_), E_int_(G_b_), E_int_(ρ_b_), E_int_(λ_||,b_), and E_int_(d(X···X)) relationships, all of them being nonlinear (R^2^ = 0.90–0.96, MAD = 0.18–0.50 kcal/mol). Quality of these relationships is better for the Cl···Cl structures compared to the Br···Br and I···I ones. Total energy density behaves well only for the I···I structures while the −E_int_(H_b_) function is not well-defined for the Br···Br structures. Laplacian ∇^2^ρ_b_ works well for the Cl···Cl and Br···Br series but not for the I···I one.

Third, the kinetic energy density at BCP can be recommended as the best estimator of E_int_ due to lower dispersion of the E_int_(G_b_) functions.

Fourth, typically, there are no statistically meaningful trends describing the series [(A)*_n_*Y–X···X–H], [(A)*_n_*Y–X···X–F], and [F–X···X–Z(B)*_m_*].

Fifth, quality of correlations for the “small” series is usually worse than that for the “large” series Cl···Cl, Br···Br, and I···I in terms of R^2^ but it is better in terms of MAD (with exception of the [F–Br···Br–Z(B)*_m_*] and [F–I···I–Z(B)*_m_*] structures).

Sixth, the obtained here E_int_(V_b_) and E_int_(G_b_) relationships are close to Equations (1)–(5) only for the [(A)*_n_*Y–Cl···Cl–H] structures. Additionally, the E_int_(property) correlations obtained here for the Cl···Cl and Br···Br bonds are significantly different from those found previously for the Cl···Cl^−^ and Br···Br^−^ interactions between the neutral fragments (A)*_n_*Y–Hal and the halide anion Hal^−^ [92]. This once again demonstrates that these dependencies are not universal and they should be established for each particular type of non-covalent interactions.

Seventh, the IAS integral of electronic virial $\iint_{IAS}^{}V\left(r\right)dr$ cannot be recommended as an E_int_ estimator at least for the Cl···Cl bond.

Eighth, the BSSE effect is insignificant for the Cl···Cl structures but becomes important for the I···I and, in particular, Br···Br structures.

The E_int_(property) relationships recommended for the Cl···Cl, Br···Br, and I···I interactions are given in Table 1 (for the whole set and “large” series”) and Table 2 (for the “small” series). Two directions toward the further extension of investigations in this field can be mentioned, i.e., (*i*) establishment of the E_int_(property) relationships for the hetero-halogen bonds Hal_1_···Hal_2_ (Hal_1_ ≠ Hal_2_) and other types of the non-covalent interactions (chalcogen, pnictogen, tetrel bonds, metallophilic interactions, etc.) and (*ii*) analysis of the effect of computational method, basis set and effective core pseudopotentials on these correlations. The corresponding studies are currently underway.

## Figures and Tables

**Table 1:** Relationships recommended for the estimates of E_int_ (in kcal/mol) for the whole set and “large” series of structures [(A)*_n_*Y–X···X–Z(B)*_m_*] at the M06-2X/6-31+G* (X = Cl, Br) and M06-2X/DZP (X = I) levels of theory.

Series	Estimator	Equation
Whole set	G_b_, kcal/(mol•bohr^3^)	−E_int_ = 0.128G_b_^2^ − 0.824G_b_ + 1.66
Cl···Cl	V_b_, kcal/(mol•bohr^3^)	−E_int_ = 0.1006V_b_^2^ + 0.218V_b_ + 0.55
	G_b_, kcal/(mol•bohr^3^)	−E_int_ = 0.0841G_b_^2^ − 0.367G_b_ + 0.84
	ρ_b_, e/Å^3^	−E_int_ = 535.9ρ_b_^2^ − 31.13ρ_b_ + 0.87
	∇^2^ρ_b_, e/Å^5^	−E_int_ = 0.09e^2.405^^∇^^2^^ρ^^b^ + 0.17
	λ_||,b_, e/Å^5^	−E_int_ = 0.099e^1.762λ||,b^ + 0.17
	d(X···X), Å	−E_int_ = 3.66×10^9^e^−6.98d(X···X)^ + 0.43
Br···Br	V_b_, kcal/(mol•bohr^3^)	−E_int_ = 0.0926V_b_^2^ + 0.173V_b_ + 0.16
	G_b_, kcal/(mol•bohr^3^)	−E_int_ = 0.1178G_b_^2^ − 0.73G_b_ + 1.50
	ρ_b_, e/Å^3^	−E_int_ = 380.6ρ_b_^2^ − 24.78ρ_b_ + 0.42
	∇^2^ρ_b_, e/Å^5^	−E_int_ = 0.07e^2.624^^∇^^2^^ρ^^b^ − 0.10
	λ_||,b_, e/Å^5^	−E_int_ = 0.30e^1.306λ||,b^ − 0.71
	d(X···X), Å	−E_int_ = 1.05×10^9^e^−6.35d(X···X)^ + 0.19
I···I	V_b_, kcal/(mol•bohr^3^)	−E_int_ = 0.0635V_b_^2^ + 0.217V_b_ + 0.25
	G_b_, kcal/(mol•bohr^3^)	−E_int_ = 0.1564G_b_^2^ − 1.138G_b_ + 2.25
	ρ_b_, e/Å^3^	−E_int_ = 305.2ρ_b_^2^ − 21.78ρ_b_ + 0.44
	λ_||,b_, e/Å^5^	−E_int_ = 0.031e^2.818λ||,b^ − 0.25
	H_b_, kcal/(mol•bohr^3^)	−E_int_ = −2.35H_b_ + 1.87
	d(X···X), Å	−E_int_ = 1.03×10^8^e^−5.15d(X···X)^ + 0.07

**Table 2:** Relationships −E_int_(V_b_) recommended for the estimates of E_int_ (in kcal/mol) for the “small” series of structures [(A)*_n_*Y–X···X–H] and [(A)*_n_*Y–X···X–F] at the M06-2X/6-31+G* (X = Cl, Br) and M06-2X/DZP (X = I) levels of theory [V_b_ in kcal/(mol•bohr^3^)].

Series	Equation
[(A)*_n_*Y–Cl···Cl–H]	−E_int_ = −0.47V_b_ − 0.27
[(A)*_n_*Y–Cl···Cl–F]	−E_int_ = −0.21V_b_ + 0.07
[(A)*_n_*Y–Br···Br–H]	−E_int_ = −0.79V_b_ − 1.61
[(A)*_n_*Y–Br···Br–F]	−E_int_ = −0.49V_b_ − 1.22
[(A)*_n_*Y–I···I–H]	−E_int_ = 0.0581V_b_^2^ + 0.007V_b_ − 0.19
[(A)*_n_*Y–I···I–F]	−E_int_ = 0.0360V_b_^2^ + 0.025V_b_ − 0.31

## Supplementary Materials

The following are available online at https://www.mdpi.com/1420-3049/24/15/2733/s1, discussion of the BSSE effect, Figure S1: plots of −E_int_ against −V_b_, G_b_, ρ_b_, ∇^2^ρ_b_, λ_||,b_, H_b_ and d(X···X) for the series [(A)*_n_*Y–X···X–H], [(A)*_n_*Y–X···X–F] and [F–X···X–Z(B)*_m_*], Figure S2: plots of −E_int_ against G_b_, ρ_b_, ∇^2^ρ_b_, λ_||,b_ and d(X···X) for the “small” series of structures [(A)*_n_*Y–X···X–Z(B)*_m_*], Figure S3: plots of −E_int_ with the CP correction against −E_int_ without the CP correction and plots of −E_int_ with and without the CP correction against −V_b_ for the “large” series of structures [(A)*_n_*Y–X···X–Z(B)*_m_*], Table S1: calculated structures [(A)*_n_*Y–X···X–Z(B)*_m_*], Table S2: negative X···X interaction energies, −E_int_, with BSSE correction calculated for the [(A)*_n_*Y–X...X–Z(B)*_m_*] structures at the M06-2X/6-31+G* (X = Cl, Br) and M06-2X/DZP (X = I) levels.
