# Supplementary material for: Relationships between Interaction Energy and Electron Density Properties for Homo Halogen Bonds of the [(A)nY–X···X–Z(B)m] Type (X = Cl, Br, I)
Source: Molecules. 2019 Jul 27;24(15):2733. doi: 10.3390/molecules24152733 (PMC6695802; doi:10.3390/molecules24152733)
Supplement: Supplementary file 1 [file molecules-24-02733-s001.zip › supportesp22.pdf]

Relationships between interaction energy and electron density properties for homo-halogen bonds of the [(A)$_n$Y–X···X–Z(B)$_m$] type (X = Cl, Br, I)

Maxim L. Kuznetsov

Centro de Química Estrutural, Instituto Superior Técnico, Universidade de Lisboa, Avenida Rovisco Pais, Lisboa 1049-001, Portugal; max@mail.ist.utl.pt

Supplementary Material

Effect of BSSE

The effect of basis set superposition error may play a very important role, in particular, for relatively small basis sets. As can be seen from Figure S3,A–C, the BSSE effect is insignificant for structures of the Cl···Cl type, E_{int} values with and without CP correction being similar. Three points corresponding to structures bearing the heavier Br atom (Br–Cl···Cl–H, Br–Cl···Cl–F, and F–Cl···Cl–Br) are out of the trend. For the I···I structures, there is also a very good correlation of $E_{int}^{with\ CP}$ against $E_{int}^{without\ CP}$. However, the BSSE effect here is significant with the slope of the correlation being 0.78 and the intercept of –1.61. For the Br···Br structures, there is no reasonable correlation between E_{int} with and without CP correction demonstrating the extreme importance of BSSE for the structures of this type for the 6-31+G* basis set. Such different behavior of the Br···Br and I···I structures may be accounted for by the different basis sets used for their calculations (6-31+G* and DZP, respectively).

In accord with the CP correction values, the $E_{int}(V_b)$ relationships are very similar for both corrected and uncorrected E_{int} in the case of the Cl···Cl structures (Figure S3,D). For the I···I structures, the $-E_{int}^{with\ CP}(-V_b)$ and $-E_{int}^{without\ CP}(-V_b)$ correlations have close curvature and horizontal vertex positions but the former function has the intercept point lower by *ca.* 2 kcal/mol compared to the latter function (Figure S3,F). The $E_{int}^{without\ CP}(V_b)$ relationship for the Br···Br structures (Figure S3,E) has poor MAD value (0.78 kcal/mol) and, therefore, the BSSE correction is crucial for these types of structure and basis set.

Table S1. Calculated structures [(A)$_n$Y–X···X–Z(B)$_m$] (**A**, **B** and **C** mean structures [(A)$_n$Y–X···X–H], [(A)$_n$Y–X···X–F] and [F–X···X–Z(B)$_m$], respectively, structures included into the analysis are marked as "V", structures for which an equilibrium geometry was not found or those with not appropriate geometry are marked as "X").

(A)$_n$Y or Z(B)$_m$	X = Cl			X = Br			X = I		
	**A**	**B**	**C**	**A**	**B**	**C**	**A**	**B**	**C**
**Z = Hal**									
F–	V	V	V	V	V	V	V	V	V
Cl–	V	V	V	V	V	V	V	V	V
Br–	V	V	V	V	V	V	V	V	V
**Z = C (sp^3)**									
Me–	X	X	X	X	V	V	V	V	V
H_2(Me)C–	X	X	X	V	V	V	V	V	V
H(Me)$_2$C–	X	X	X	V	V	V	V	X	X
Me_3C–	X	X	X	V	V	V	V	X	X
H_2FC–	V	V	V	V	V	V	V	V	V
H(F)$_2$C–	V	V	V	V	V	V	V	V	V
F_3C–	V	V	V	V	V	V	V	V	V
N≡C–CH_2–	X	V	V	V	V	V	V	V	V
(N≡C)$_2$CH–	V	V	V	V	V	V	V	X	X
(N≡C)$_3$C–	V	V	V	X	V	V	V	X	V
**Z = C (sp^2)**									
H_2C=CH–	X	X	X	V	V	V	V	V	X
FCH=CH–	X	X	V	V	V	V	V	V	V
F_2C=CH–	V	X	V	V	V	V	V	V	V
F_2C=CF–	V	V	V	V	V	V	V	V	V
F_3C(H)C=CH–	X	X	V	X	V	V	V	V	V
(F_3C)$_2$C=CH–	X	X	V	X	V	V	V	V	V
H(O=)C–	X	X	X	X	V	X	V	X	V
Me(O=)C–	X	X	X	X	V	X	V	X	V
(MeO)(O=)C–	V	V	V	V	X	V	V	X	V
Ph–	X	X	V	V	V	V	V	V	V
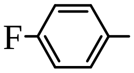	X	X	V	V	V	V	V	V	V
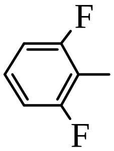	V	V	V	V	V	V	V	V	V
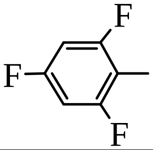	V	V	V	V	V	V	V	V	V
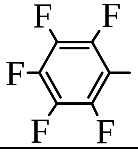	V	V	V	V	V	V	V	V	V
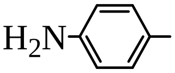	X	X	X	V	V	V	V	V	X

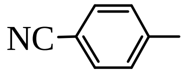	V	V	V	X	V	V	V	V	X
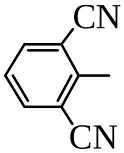	V	V	V	X	V	V	V	V	V
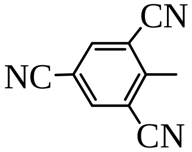	V	V	V	X	V	V	V	V	V
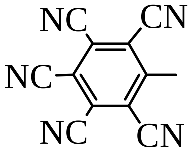	V	V	V	X	V	V	X	V	V
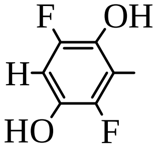	V	X	V	X	V	X	V	V	V
**Z = C (sp)**									
H–C≡C–	V	V	V	V	V	V	V	V	V
Me–C≡C–	V	V	V	V	V	V	V	V	V
F–C≡C–	V	V	V	V	V	V	V	V	V
Cl–C≡C–	V	V	V	V	V	V	V	V	V
F_3C–C≡C–	V	V	V	V	V	V	V	V	V
N≡C–	V	V	V	X	V	V	V	V	V
**Z = N**									
H_2N–	X	V	V	V	V	V	V	V	V
Me_2N–	X	X	V	V	V	V	V	V	X
H(Ph)N–	X	V	V	V	V	V	V	V	X
Ph_2N–	X	X	V	V	V	X	V	X	X
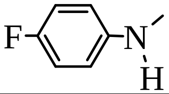	X	V	V	V	V	V	V	X	X
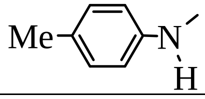	X	V	V	V	V	V	V	X	X
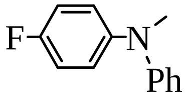	X	X	V	V	X	V	V	X	X
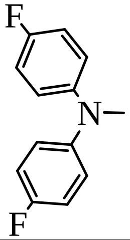	V	V	V	V	V	V	V	X	V

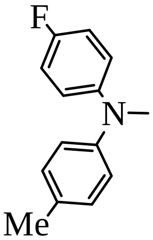	X	X	V	V	V	X	V	X	X
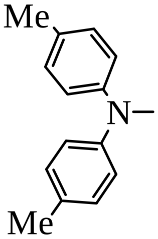	X	X	V	V	V	X	V	X	X
O=N–	X	X	X	V	V	V	V	V	X
H_2C=N–	V	V	V	V	V	V	V	V	V
F_2C=N–	V	V	V	V	V	V	V	V	V
Me_2C=N–	X	X	X	X	V	X	V	V	V
O_2N–	V	V	V	V	V	V	V	V	X
**Z = S**					V				
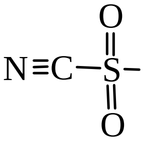	V	V	V	X	V	V	V	V	V
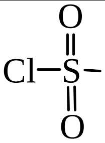	V	V	V	V	V	V	V	V	V
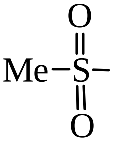	V	V	V	X	V	V	V	X	X
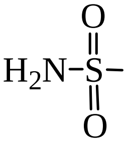	V	V	V	V	V	V	X	V	V
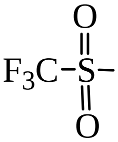	V	V	V	V	V	V	V	V	V
Me(O=)S–	X	V	X	X	V	V	V	V	X
F_3C(O=)S–	V	V	V	V	V	V	V	V	V
Cl(O=)S–	V	V	V	X	V	V	V	V	V
N≡C(O=)S–	V	V	V	V	V	V	V	X	X
**Z = P**									
H_2P–	X	X	X	X	V	V	V	V	V
H(F)P–	V	X	X	X	V	V	V	V	V
F_2P–	V	X	V	X	V	V	V	V	V
Me_2P–	X	X	X	V	X	V	V	V	V
Cl_2(O=)P–	V	V	V	V	V	V	V	V	V
F_2(O=)P–	V	V	V	V	V	V	V	V	V
H_2(O=)P–	V	X	X	X	V	X	V	V	V

H(F)(O=)P–	V	V	V	V	V	V	V	V	X
$(F_3C)_2(=O)P$–	V	V	V	X	X	X	V	V	V
**Z = B**									
F_2B–	X	V	V	X	V	X	V	V	V
H_2B–	X	V	X	X	X	V	V	V	X
H(F)B–	X	V	V	X	V	X	V	V	V
H(HO)B–	X	X	X	X	V	X	V	V	V
**Z = Si**									
Me_3Si–	X	X	X	X	X	X	V	V	V
F_3Si–	V	V	V	V	X	V	V	V	V
H_3Si–	X	V	X	X	V	X	V	V	V
$H_2(F)Si$–	X	V	X	X	V	V	V	V	V
$H(F)_2Si$–	X	V	X	V	X	V	V	V	V
**Z = O**									
HO–	V	V	V	V	V	V	V	V	V
MeO–	V	V	V	V	V	V	V	V	X
PhO–	V	V	V	V	V	V	V	V	X
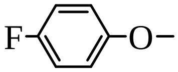	V	V	V	V	V	V	V	V	X
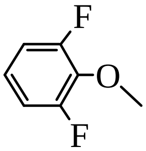	V	V	V	V	V	V	V	V	V
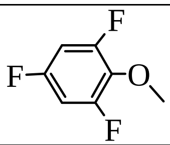	V	V	V	V	V	V	V	V	V
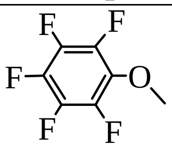	V	V	V	V	V	V	V	V	V
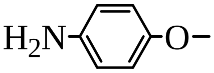	V	V	V	V	V	V	V	X	V
**Z = H**									
H	X	X	V	X	V	X	V	V	V

Table S2. Negative X•••X interaction energies, $-E_{int}$, with BSSE correction calculated for the $[(A)_nY–X...X–Z(B)_m]$ structures at the M06-2X/6-31+G* (X = Cl, Br) and M06-2X/DZP (X = I) levels.

$[(A)_nY–X...X–Z(B)_m]$	X = Cl	X = Br	X = I
F–X···X–H	2.51	3.95	4.85
Cl–X···X–H	1.47	2.43	3.45
Br–X···X–H	1.09	2.12	3.26
HO–X···X–H	1.49		
HO–X···X–H	1.47	1.94	2.26
H_2N–X···X–H		1.32	1.00
H_2N–X···X–H			
H–X···X–H			0.22
FH_2C–X···X–H	0.40	0.40	0.12
F_2HC–X···X–H	0.42	0.59	0.37
F_3C–X···X–H	0.78	0.82	0.86
HCC–X···X–H	0.78		
HCC–X···X–H	0.75	1.09	1.07
Ph–X···X–H			0.19
C_6F_5–X···X–H	0.63	0.93	1.08
H_2N–Ph–X···X–H			0.92
F–Ph–X···X–H			0.30
MeCC–X···X–H	0.76		0.78
MeCC–X···X–H	0.58	1.00	
FCC–X···X–H	0.77	1.14	1.19
MeO–X···X–H	0.97	1.73	2.03
Me_2N–X···X–H			0.43
Me_2N–X···X–H			0.52
C_6F_2OH2H–X···X–H	0.83		0.81
C_6F_2OH2H–X···X–H	0.75		
$NCSO_2$–X···X–H	1.17	1.64	2.33
$ClSO_2$–X···X–H	1.01	1.65	2.46
H_2NSO_2–X···X–H	0.48	0.84	0.96
F_3CSO_2–X···X–H	0.82	1.26	1.59
MeSO–X···X–H		0.49	
Cl_2PO–X···X–H	0.87	1.11	1.10
O_2N–X···X–H	1.26	1.95	2.42
MeOCO–X···X–H	0.47	0.56	0.33
F_3Si–X···X–H	0.42		0.32
F_3Si–X···X–H	0.41	0.58	
H_3Si–X···X–H		0.19	
H_2FSi–X···X–H		0.26	
HF_2Si–X···X–H		0.35	
HFP–X···X–H	0.26		
F_2P–X···X–H	0.38		0.02
F_2B–X···X–H		0.42	0.14
PhO–X···X–H	1.55	2.08	2.90
F–PhO–X···X–H	1.26	2.17	3.06
H_2NPh–O–X···X–H	1.18	1.94	1.91

PhNH–X···X–H		1.34	1.19
Ph_2N–X···X–H			1.40
F–PhNH–X···X–H		1.37	1.23
MePhNH–X···X–H		1.15	1.07
FPhPhN–X···X–H			1.30
p–FPh_2N–Cl...Cl–H	0.72	1.38	1.38
F-PhMe-PhN–X···X–H			1.23
$MePh_2N$–X···X–H			1.04
H_2B–X···X–H		0.20	
HFB–X···X–H		0.28	-0.05
$MeSO_2$–X···X–H	0.18	0.60	0.52
F_3CSO–X···X–H	0.58		
F_3CSO–X···X–H	0.49	0.93	0.85
NCSO–X···X–H	0.66	1.12	1.30
ClSO–X···X–H	0.51	0.90	0.83
F_2PO–X···X–H	0.74	1.16	1.18
H_2PO–X···X–H	0.18		
HFPO–X···X–H	0.53	0.66	0.43
CH_2N–X···X–H	0.45	0.76	0.73
F_2CN–X···X–H	1.19		
F_2CN–X···X–H	1.00	1.68	2.04
NC–X···X–H	1.21	1.64	1.78
FCH=CH–X···X–H			0.31
F_2CCH–X···X–H	0.43		0.71
o–F_2Ph–X···X–H	0.42	0.57	0.53
p,o–F_3Ph–Cl...Cl–H	0.49	0.65	0.66
o–F_2Ph–O–X···X–H	1.55	2.39	3.10
p,o–F_3Ph–O–Cl...Cl–H	1.59	2.45	3.30
C_6F_5–O–X···X–H	1.82	2.84	4.11
F_3C_2PO–X···X–H	0.87	1.10	0.99
ClCC–X···X–H	0.78	1.14	1.17
F_3CCC–Cl...Cl–H	1.04	1.42	1.53
F_2C=CF–X···X–H	0.75		
F_2CCF–X···X–H	0.70	0.95	1.00
$NCCH_2$–X···X–H		0.73	0.75
NC_2CH–X···X–H	1.00	1.48	1.74
NC_3C–X···X–H	1.36	2.02	2.99
F_3CCH=CH–X···X–H			0.59
F_3C_2C=CH–X···X–H			1.12
p–NCPh–X···X–H	0.50	0.57	0.46
o–NC_2Ph–X···X–H	0.61	0.92	1.14
p,o–NC_3Ph–X···X–H	0.78	0.92	1.24
NC_5Ph–X···X–H	1.10	1.44	2.02
F–X···X–F	1.10	2.32	2.47
Cl–X···X–F	0.71	1.25	1.63
Br–X···X–F	0.40	1.08	1.55
HO–X···X–F	0.73	1.25	1.17
H_2N–X···X–F	0.35	0.54	0.08
H–X···X–F		0.25	

Me–X···X–F		0.22	-0.13
FH_2C–X···X–F	0.25	0.04	-0.20
FH_2C–X···X–F			0.03
F_2HC–X···X–F	0.39	0.21	0.13
F_3C–X···X–F	0.31	0.22	0.19
MeH_2C–X···X–F	0.40	0.21	-0.23
Me_2HC–X···X–F	0.39	0.24	-0.30
Me_3C–X···X–F	0.38	0.25	-0.34
CH_2=CH–X···X–F	0.37	0.18	-0.12
HCC–X···X–F	0.38	0.36	0.24
Ph–X···X–F	0.34	0.25	-0.11
C_6F_5–X···X–F	0.40		
C_6F_5–X···X–F	0.29	0.31	0.23
H_2N–Ph–X···X–F	0.33	0.29	-0.06
F–Ph–X···X–F	0.35	0.25	-0.03
CHO–X···X–F		0.16	
CMeO–X···X–F		0.29	
MeCC–X···X–F	0.34	0.36	0.22
FCC–X···X–F	0.33	0.37	0.31
MeO–X···X–F	0.65	1.11	0.97
Me_2N–X···X–F	0.53	0.65	0.12
C_6OH_2F2H–X···X–F		0.38	
$NCSO_2$–X···X–F		0.54	0.81
$ClSO_2$–X···X–F	0.43	0.50	0.89
H_2NSO_2–X···X–F	0.45	0.36	0.35
F_3CSO_2–X···X–F	0.37	0.36	0.55
MeSO–X···X–F		0.34	-0.04
Cl_2PO–X···X–F	0.38	0.25	0.08
O_2N–X···X–F	0.56	0.88	0.91
MeOCO–X···X–F	0.26		-0.13
F_3Si–X···X–F	0.20		-0.23
H_3Si–X···X–F		0.16	
H_2FSi–X···X–F		0.04	-0.14
HF_2Si–X···X–F	0.18		-0.04
H_2P–X···X–F		0.34	-0.09
HFP–X···X–F		0.32	-0.08
F_2P–X···X–F		0.16	-0.02
F_2B–X···X–F		0.16	
F_2B–X···X–F		0.04	
Ph–O–X···X–F	0.68	1.23	1.40
F–PhO–X···X–F	0.71	1.11	1.27
H_2N–Ph–O–X···X–F	0.57	0.96	0.83
PhNH–X···X–F	0.43	0.49	0.33
Ph_2N–X···X–F	0.43	0.65	
F–PhNH–X···X–F	0.44	0.47	0.37
MePhNH–X···X–F	0.47	0.49	0.32
FPhPhN–X···X–F	0.42		0.33
FPh_2N–X···X–F	0.42	0.61	0.36
FPhMePhN–X···X–F	0.39	0.61	

$MePh_2N$–X⋯X–F	0.42	0.63	
H_2B–X⋯X–F			-0.29
HFB–X⋯X–F		0.06	
HOHB–X⋯X–F		-0.08	
$MeSO_2$–X⋯X–F		0.05	0.47
F_3CSO–X⋯X–F	0.53	0.42	0.34
NCSO–X⋯X–F	0.58	0.59	0.59
ClSO–X⋯X–F		0.46	0.36
F_2PO–X⋯X–F	0.20	0.17	0.19
H_2PO–X⋯X–F		0.42	
HFPO–X⋯X–F	0.29	0.01	-0.26
Me_2P–X⋯X–F	0.45		-0.22
ON–X⋯X–F	0.39	0.26	-0.20
H_2CN–X⋯X–F	0.30	0.36	0.20
F_2C=N–X⋯X–F	0.44	0.71	0.69
Me_2C=N–X⋯X–F		0.25	
NC–X⋯X–F		0.56	0.49
CHF=CH–X⋯X–F	0.44	0.32	0.09
F_2C=CH–X⋯X–F	0.30	0.22	0.17
o–F_2Ph–X⋯X–F	0.37	0.31	0.30
p,o-F_3Ph–X⋯X–F	0.35	0.32	0.21
o–F_2Ph–O–X⋯X–F	0.61	1.10	1.33
p,o–F_3Ph–O–X⋯X–F	0.73	1.29	1.59
C_6F_5O–X⋯X–F	0.81	1.50	1.92
ClCC–X⋯X–F	0.33	0.32	0.28
F_3CCC–X⋯X–F	0.32	0.47	0.42
F_2C=CF–X⋯X–F	0.30	0.27	0.13
$NCCH_2$–X⋯X–F	0.49	0.32	0.05
NC_2CH–X⋯X–F			0.47
NC_2CH–X⋯X–F	0.42	0.48	0.48
NC_3C–X⋯X–F		0.83	0.91
F_3CCH=CH–X⋯X–F		0.24	0.39
F_3C_2C=CH–X⋯X–F		0.45	0.39
p–NCPh–X⋯X–F		0.22	0.02
o–NC_2Ph–X⋯X–F		0.32	0.21
p,o–NC_3Ph–X⋯X–F		0.33	0.30
NC_5Ph–X⋯X–F		0.61	0.46
F–X⋯X–F	1.10	2.32	2.47
F–X⋯X–Cl	1.68	3.03	3.20
F–X⋯X–Br	1.89	3.02	3.50
F–X⋯X–OH	2.48	4.22	4.62
F–X⋯X–NH_2	3.38	5.19	6.27
F–X⋯X–H	2.51	3.95	4.84
F–X⋯X–Me	4.07	6.27	7.31
F–X⋯X–CH_2F	2.98	5.10	5.84
F–X⋯X–CHF_2	2.24	4.05	4.64
F–X⋯X–CF_3	1.42	2.77	3.44
F–X⋯X–CH_2Me	4.24	6.45	7.83
F–X⋯X–$CHMe_2$	4.73		

F–X···X–CMe_3	5.01		
F–X···X–CH=CH_2	3.16	4.99	
F–X···X–CCH	1.49	2.72	3.20
F–X···X–Ph	3.39	5.08	6.25
F–X···X–C_6F_5	2.15	3.01	3.68
F–X···X–$PhNH_2$	3.84	5.73	
F–X···X–PhF	3.20	4.79	5.86
F–X···X–CHO	2.52		5.45
F–X···X–CMeO	2.93		6.81
F–X···X–CCMe	1.98	3.47	4.41
F–X···X–CCF	1.54	2.81	3.24
F–X···X–OMe	3.08	4.94	
F–X···X–NMe_2	3.95	5.83	
F–X···X–$C_6F_2OH_2H$	2.24	3.03	3.86
F–X···X–SO_2CN	0.56	1.48	1.51
F–X···X–SO_2Cl	1.33	2.27	2.38
F–X···X–SO_2NH_2		3.50	4.26
F–X···X–SO_2CF_3	1.30	2.34	2.26
F–X···X–SMeO	3.69	5.86	
F–X···X–POCl2	1.30	2.40	2.60
F–X···X–NO_2	1.40	2.34	
F–X···X–COOMe	2.65		6.02
F–X···X–SiF_3	1.34	2.29	2.00
F–X···X–$SiMe_3$	4.79	7.10	9.24
F–X···X–SiH_3	3.34	5.24	6.35
F–X···X–SiH_2F	2.56	4.10	4.79
F–X···X–$SiHF_2$	2.02	2.92	3.14
F–X···X–PH_2	3.28	5.28	6.18
F–X···X–PHF	2.62	4.32	5.17
F–X···X–PF_2	2.06	3.89	4.53
F–X···X–BF_2	1.36	2.53	2.72
F–X···X–O–Ph	2.82	3.98	
F–X···X–O–PhF	2.96	3.76	
F–X···X–O–$PhNH_2$	3.52		7.48
F–X···X–NHPh	3.55	5.52	
F–X···X–NPh_2	3.71		
F–X···X–NHPhF	3.60		
F–X···X–NHPhMe	3.74		
F–X···X–NPhPhF	3.61		
F–X···X–$NPhF_2$	3.48		6.32
F–X···X–NPhFPhMe	3.65		
F–X···X–$NPhMe_2$	3.88		
F–X···X–BH_2	1.98	3.67	
F–X···X–BHF	2.35	3.53	4.92
F–X···X–BHOH	2.80	4.30	5.55
F–X···X–SO_2Me	2.73		
F–X···X–$SOCF_3$	2.11	3.27	4.00
F–X···X–SOCN	1.74		
F–X···X–SOCl	2.27	3.89	4.53

F–X···X–POF_2	0.69	1.56	1.40
F–X···X–POH_2			5.38
F–X···X–POH_2	2.13	3.63	4.25
F–X···X–POHF	1.50	2.86	
F–X···X–PMe_2	4.17	6.30	7.79
F–X···X–NO	3.73	6.03	
F–X···X–NCH_2	3.20	5.31	5.89
F–X···X–N=CF_2	2.22	3.68	3.88
F–X···X–N=CMe_2	3.58	5.46	7.25
F–X···X–CN	0.20	0.94	1.13
F–X···X–CH=CHF	2.98	4.57	5.87
F–X···X–CH=CF_2	2.68	4.12	4.89
F–X···X–PhF_2–o	2.79	4.08	5.15
F–X···X–PhF_3–p,o	2.65	3.75	4.81
F–X···X–O–PhF_2–o	2.38	3.30	4.64
F–X···X–O–PhF_3–p,o	2.04	3.02	4.43
F–X···X–O–C_6F_5	1.63	2.42	2.89
F–X···X–$PO(CF_3)_2$	0.86	1.54	1.70
F–X···X–CCCl	1.39	2.63	3.25
F–X···X–$CCCF_3$	0.65	1.58	1.90
F–X···X–CF=CF_2	1.80	2.88	3.33
F–X···X–CH_2CN	2.78	4.31	5.07
F–X···X–$CHCN_2$	1.96		
F–X···X–CCN_3	1.24		1.49
F–X···X–CH=$CHCF_3$	2.43	3.83	4.29
F–X···X–CH=$C(CF_3)_2$	1.74	2.69	3.14
F–X···X–PhCN–p	2.73	4.00	
F–X···X–$PhCN_2$–o	2.23	3.26	3.75
F–X···X–$PhCN_3$–p,o	1.81	2.56	2.62
F–X···X–$PhCN_5$		1.82	1.37

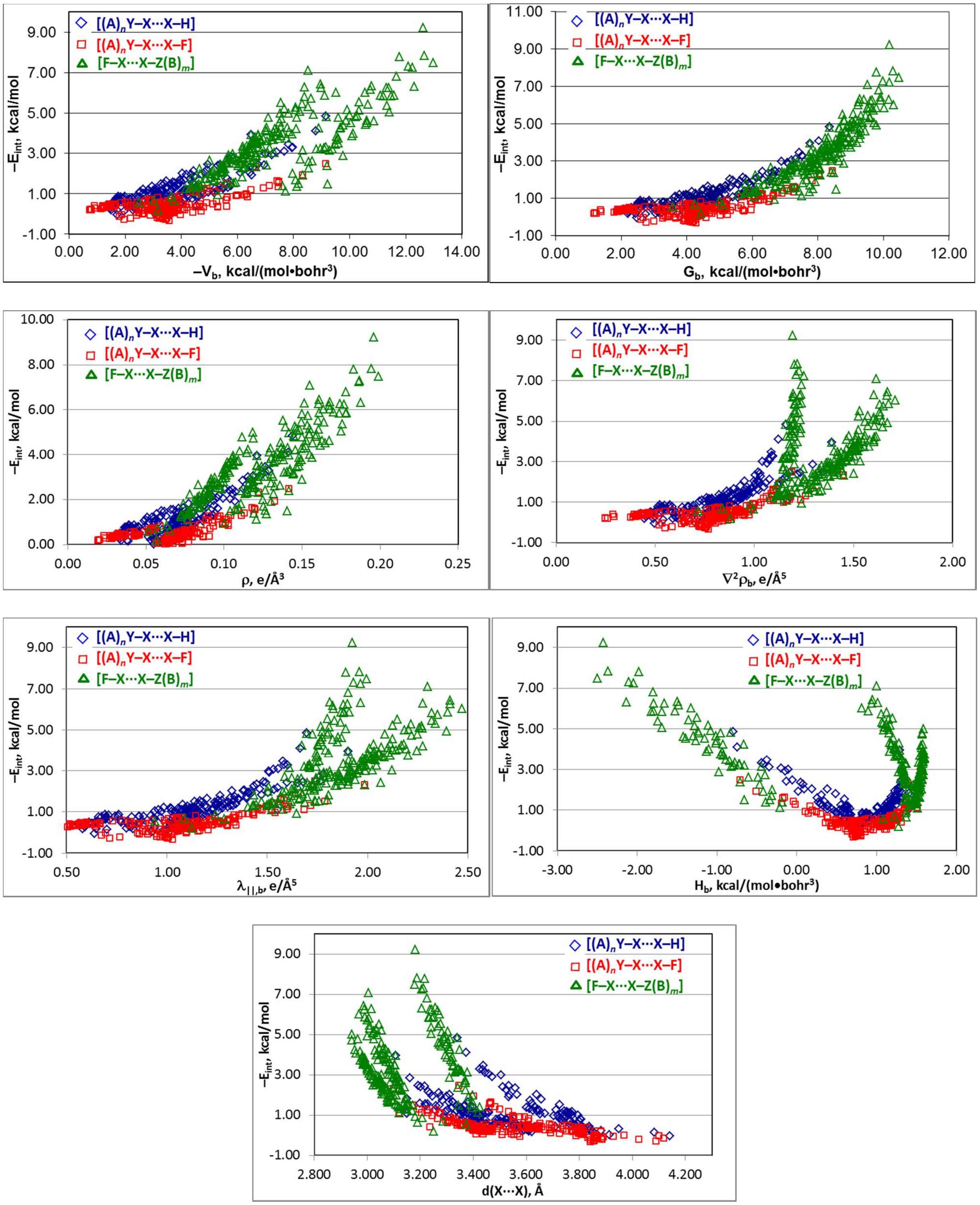

Figure S1. Plots of $-E_{int}$ against $-V_b$, G_b, ρ_b, $\nabla^2\rho_b$, $\lambda_{\|,b}$, H_b and d(X···X) for the series [(A)$_n$Y–X···X–H], [(A)$_n$Y–X···X–F] and [F–X···X–Z(B)$_m$].

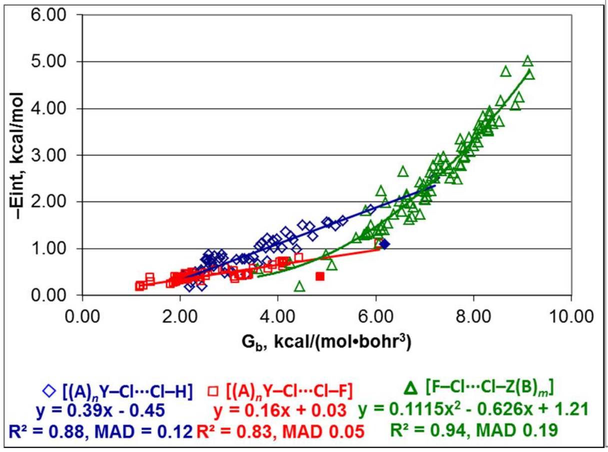
−Eint, kcal/mol
G_b, kcal/(mol•bohr³)
[(A)_nY–Cl···Cl–H]
y = 0.39x - 0.45
R² = 0.88, MAD = 0.12
[(A)_nY–Cl···Cl–F]
y = 0.16x + 0.03
R² = 0.83, MAD 0.05
[F–Cl···Cl–Z(B)_m]
y = 0.1115x² - 0.626x + 1.21
R² = 0.94, MAD 0.19

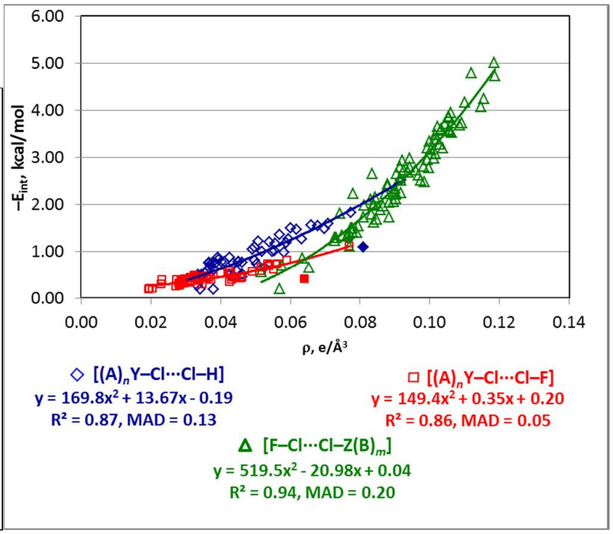
−E_int, kcal/mol
ρ, e/Å³
[(A)_nY–Cl···Cl–H]
y = 169.8x² + 13.67x - 0.19
R² = 0.87, MAD = 0.13
[(A)_nY–Cl···Cl–F]
y = 149.4x² + 0.35x + 0.20
R² = 0.86, MAD = 0.05
[F–Cl···Cl–Z(B)_m]
y = 519.5x² - 20.98x + 0.04
R² = 0.94, MAD = 0.20

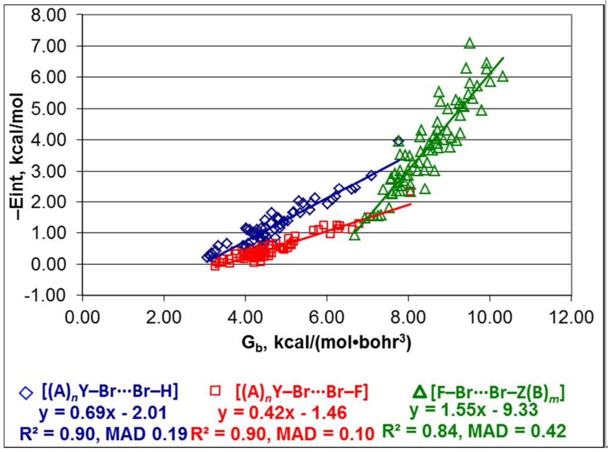
−Eint, kcal/mol
G_b, kcal/(mol•bohr³)
[(A)_nY–Br···Br–H]
y = 0.69x - 2.01
R² = 0.90, MAD 0.19
[(A)_nY–Br···Br–F]
y = 0.42x - 1.46
R² = 0.90, MAD = 0.10
[F–Br···Br–Z(B)_m]
y = 1.55x - 9.33
R² = 0.84, MAD = 0.42

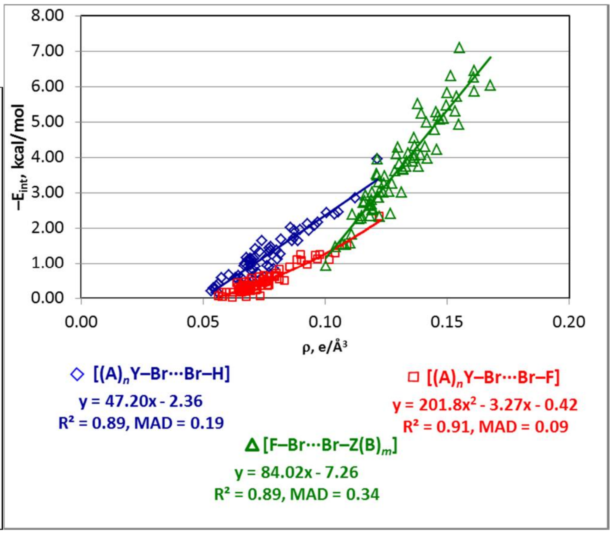
−E_int, kcal/mol
ρ, e/Å³
[(A)_nY–Br···Br–H]
y = 47.20x - 2.36
R² = 0.89, MAD = 0.19
[(A)_nY–Br···Br–F]
y = 201.8x² - 3.27x - 0.42
R² = 0.91, MAD = 0.09
[F–Br···Br–Z(B)_m]
y = 84.02x - 7.26
R² = 0.89, MAD = 0.34

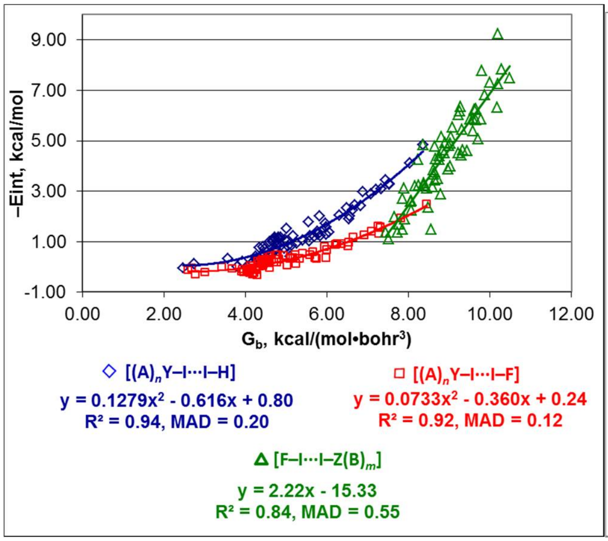
−Eint, kcal/mol
G_b, kcal/(mol•bohr³)
[(A)_nY–I···I–H]
y = 0.1279x² - 0.616x + 0.80
R² = 0.94, MAD = 0.20
[(A)_nY–I···I–F]
y = 0.0733x² - 0.360x + 0.24
R² = 0.92, MAD = 0.12
[F–I···I–Z(B)_m]
y = 2.22x - 15.33
R² = 0.84, MAD = 0.55

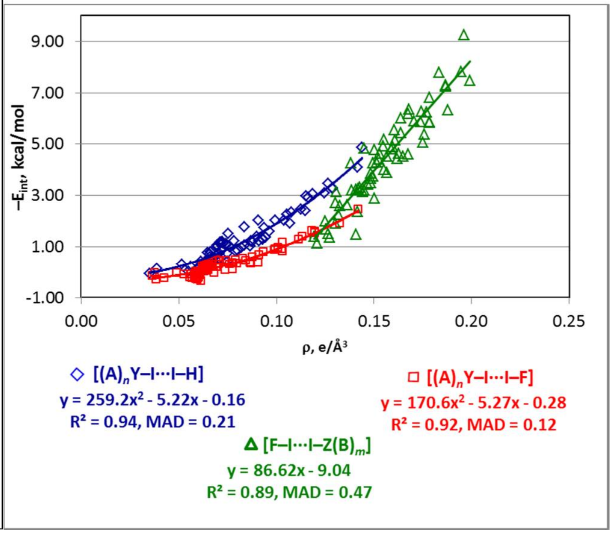
−E_int, kcal/mol
ρ, e/Å³
[(A)_nY–I···I–H]
y = 259.2x² - 5.22x - 0.16
R² = 0.94, MAD = 0.21
[(A)_nY–I···I–F]
y = 170.6x² - 5.27x - 0.28
R² = 0.92, MAD = 0.12
[F–I···I–Z(B)_m]
y = 86.62x - 9.04
R² = 0.89, MAD = 0.47

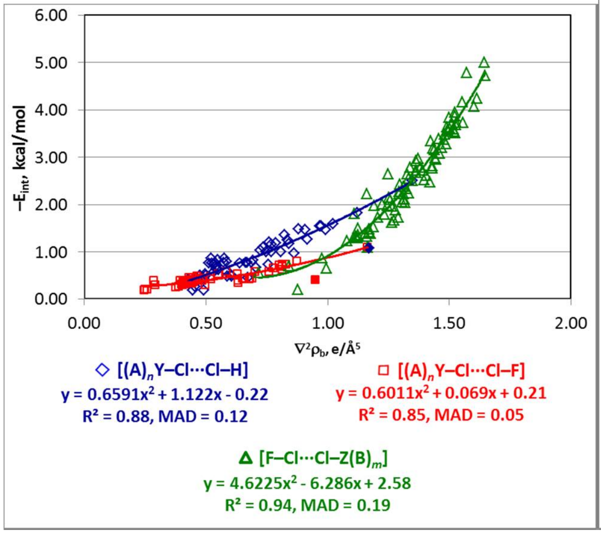
−E_int, kcal/mol
∇²ρ_b, e/Å⁵
◇ [(A)_nY–Cl···Cl–H]
y = 0.6591x² + 1.122x - 0.22
R² = 0.88, MAD = 0.12
□ [(A)_nY–Cl···Cl–F]
y = 0.6011x² + 0.069x + 0.21
R² = 0.85, MAD = 0.05
Δ [F–Cl···Cl–Z(B)_m]
y = 4.6225x² - 6.286x + 2.58
R² = 0.94, MAD = 0.19

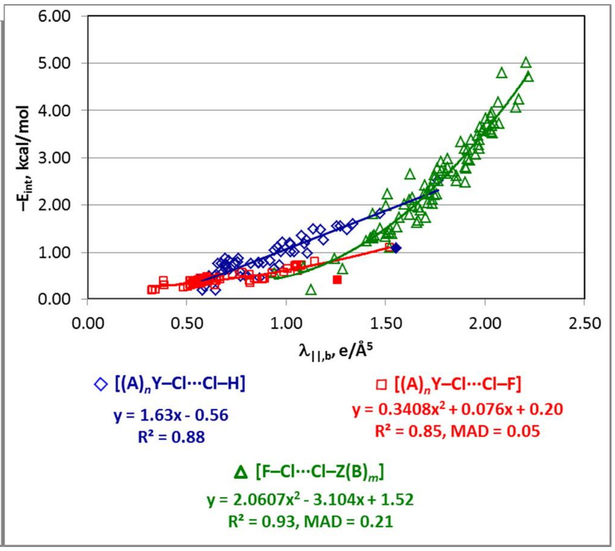
−E_int, kcal/mol
λ_||,b, e/Å⁵
◇ [(A)_nY–Cl···Cl–H]
y = 1.63x - 0.56
R² = 0.88
□ [(A)_nY–Cl···Cl–F]
y = 0.3408x² + 0.076x + 0.20
R² = 0.85, MAD = 0.05
Δ [F–Cl···Cl–Z(B)_m]
y = 2.0607x² - 3.104x + 1.52
R² = 0.93, MAD = 0.21

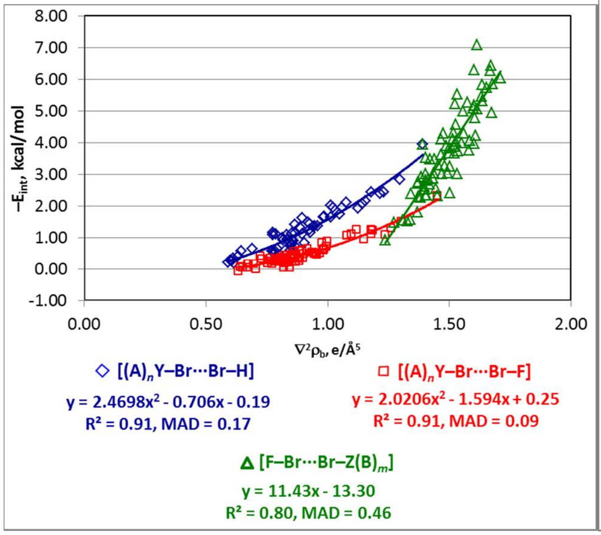
−E_int, kcal/mol
∇²ρ_b, e/Å⁵
◇ [(A)_nY–Br···Br–H]
y = 2.4698x² - 0.706x - 0.19
R² = 0.91, MAD = 0.17
□ [(A)_nY–Br···Br–F]
y = 2.0206x² - 1.594x + 0.25
R² = 0.91, MAD = 0.09
Δ [F–Br···Br–Z(B)_m]
y = 11.43x - 13.30
R² = 0.80, MAD = 0.46

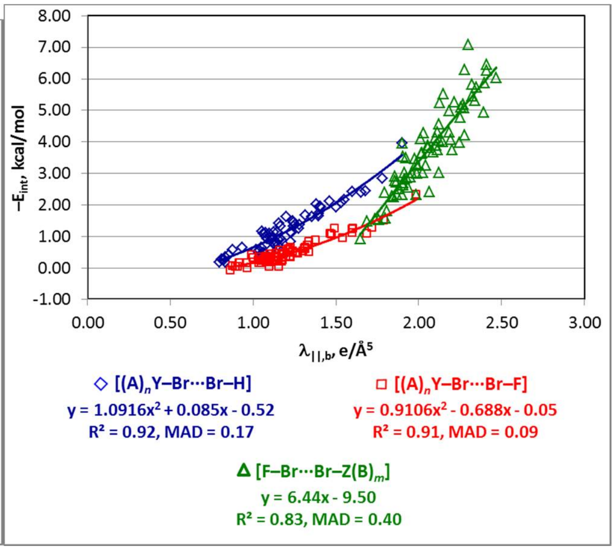
−E_int, kcal/mol
λ_||,b, e/Å⁵
◇ [(A)_nY–Br···Br–H]
y = 1.0916x² + 0.085x - 0.52
R² = 0.92, MAD = 0.17
□ [(A)_nY–Br···Br–F]
y = 0.9106x² - 0.688x - 0.05
R² = 0.91, MAD = 0.09
Δ [F–Br···Br–Z(B)_m]
y = 6.44x - 9.50
R² = 0.83, MAD = 0.40

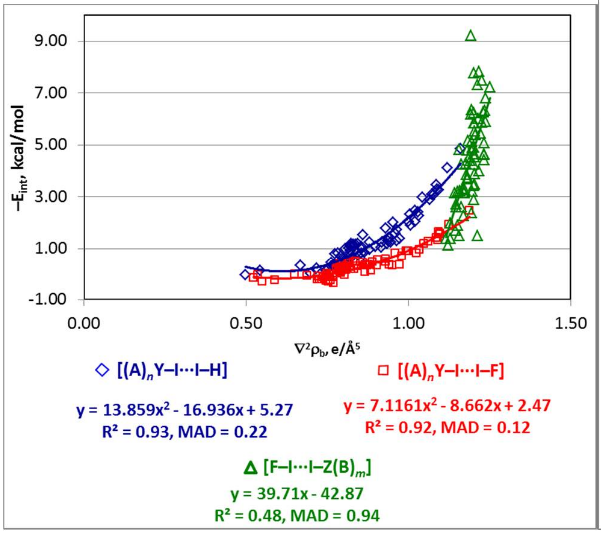
−E_int, kcal/mol
∇²ρ_b, e/Å⁵
◇ [(A)_nY–I···I–H]
y = 13.859x² - 16.936x + 5.27
R² = 0.93, MAD = 0.22
□ [(A)_nY–I···I–F]
y = 7.1161x² - 8.662x + 2.47
R² = 0.92, MAD = 0.12
Δ [F–I···I–Z(B)_m]
y = 39.71x - 42.87
R² = 0.48, MAD = 0.94

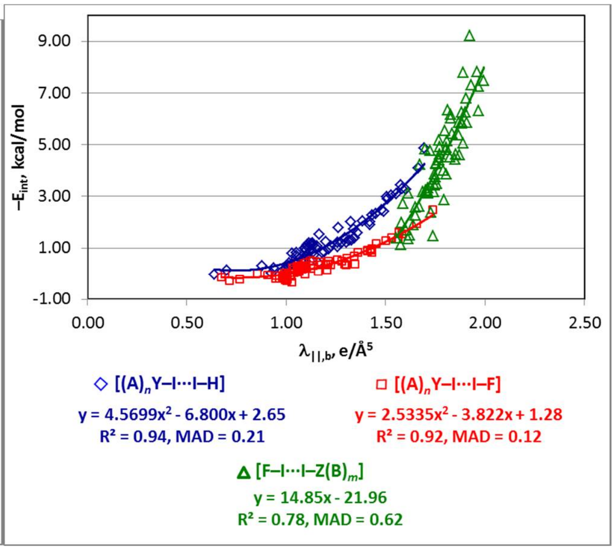
−E_int, kcal/mol
λ_||,b, e/Å⁵
◇ [(A)_nY–I···I–H]
y = 4.5699x² - 6.800x + 2.65
R² = 0.94, MAD = 0.21
□ [(A)_nY–I···I–F]
y = 2.5335x² - 3.822x + 1.28
R² = 0.92, MAD = 0.12
Δ [F–I···I–Z(B)_m]
y = 14.85x - 21.96
R² = 0.78, MAD = 0.62

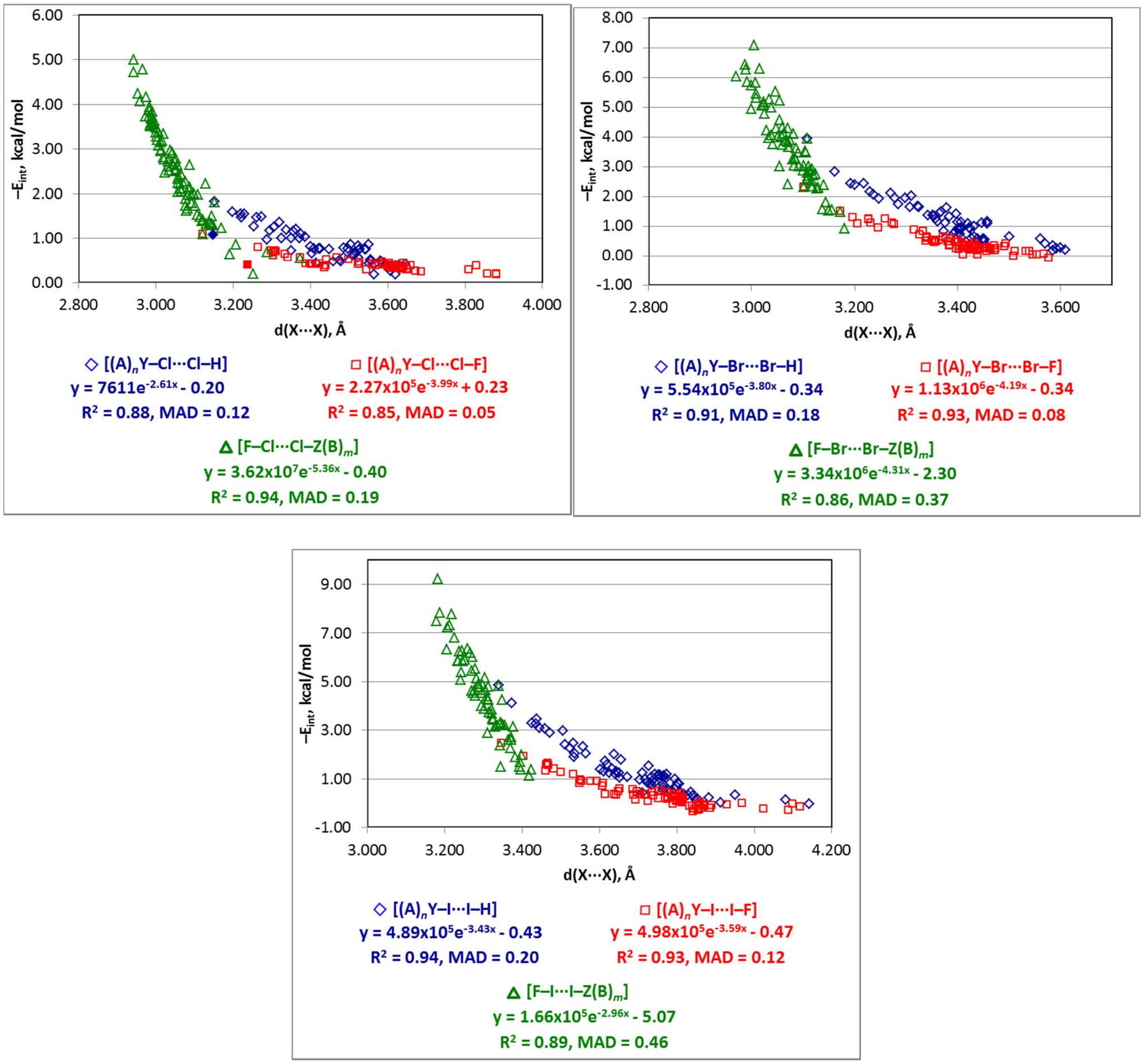

Figure S2. Plots of $-E_{int}$ against G_b, ρ_b, $\nabla^2\rho_b$, $\lambda_{\|,b}$ and d(X···X) for the "small" series of structures $[(A)_nY–X\cdots X–Z(B)_m]$ (structures Br–Cl···Cl–H and Br–Cl···Cl–F are not included in the fittings).

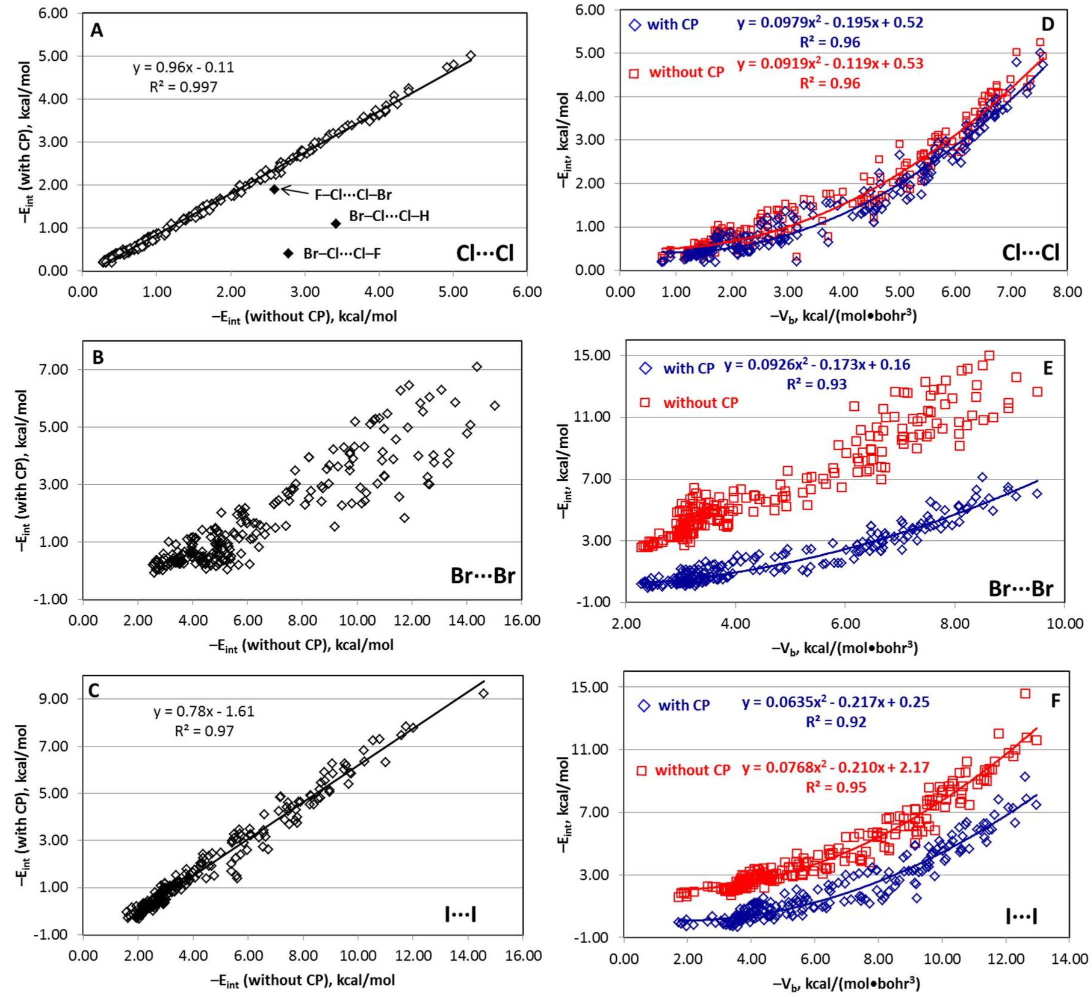

Figure S3. Plots of $–E_{int}$ with the CP correction against $–E_{int}$ without the CP correction (**A**–**C**) and plots of $–E_{int}$ with and without the CP correction against $–V_b$ (**D**–**F**) for the "large" series of structures [(A)$_n$Y–X···X–Z(B)$_m$]. Points corresponding to structures Br–Cl···Cl–H, Br–Cl···Cl–F, and F–Cl···Cl–Br are not included in the fittings in parts **A** and **D**.
